# Analysis and visualisation of electronic health records data to identify undiagnosed patients with rare genetic diseases

**DOI:** 10.1038/s41598-024-55424-8

**Published:** 2024-03-01

**Authors:** Daniel Moynihan, Sean Monaco, Teck Wah Ting, Kaavya Narasimhalu, Jenny Hsieh, Sylvia Kam, Jiin Ying Lim, Weng Khong Lim, Sonia Davila, Yasmin Bylstra, Iswaree Devi Balakrishnan, Mark Heng, Elian Chia, Khung Keong Yeo, Bee Keow Goh, Ritu Gupta, Tele Tan, Gareth Baynam, Saumya Shekhar Jamuar

**Affiliations:** 1 Curtin University, Perth, Australia; 2 Health Catalyst, Utah, USA; 3 Genetics Service, Department of Paediatrics, KK Women’s and Children’s Hospital, 100 Bukit Timah Road, Singapore, 229899 Singapore; 4 SingHealth Duke-NUS Genomic Medicine Centre, Singapore, Singapore; 5 Department of Neurology, National Neuroscience Institute (Singapore General Hospital), Singapore, Singapore; 6 Department of Internal Medicine, Singapore General Hospital, Singapore, Singapore; 7 SingHealth Duke-NUS Institute of Precision Medicine, Singapore, Singapore; 8 Cancer & Stem Cell Biology Program, Duke-NUS Medical School, Singapore, Singapore; 9 Laboratory of Genome Variation Analytics, Genome Institute of Singapore, Singapore, Singapore; 10 National Heart Centre Singapore, Singapore, Singapore; 11 SingHealth Office of Insights and Analytics, Singapore, Singapore; 12 Data Analytics Office, KK Women’s and Children’s Hospital, Singapore, Singapore; 13 Rare Care Centre, Perth Children’s Hospital, Perth, WA Australia; 14 Western Australian Register of Developmental Anomalies, Perth, WA Australia

**Keywords:** Clinical genetics, Data mining

## Abstract

Rare genetic diseases affect 5–8% of the population but are often undiagnosed or misdiagnosed. Electronic health records (EHR) contain large amounts of data, which provide opportunities for analysing and mining. Data analysis in the form of visualisation and statistical testing, was performed on a database containing deidentified health records of 1.28 million patients across 3 major hospitals in Singapore, in a bid to improve the diagnostic process for patients who are living with an undiagnosed rare disease, specifically focusing on Fabry Disease and Familial Hypercholesterolaemia (FH). On a baseline of 4 patients, we identified 2 additional patients with potential diagnosis of Fabry disease, suggesting a potential 50% increase in diagnosis. Similarly, we identified > 12,000 individuals who fulfil the clinical and laboratory criteria for FH but had not been diagnosed previously. This proof-of-concept study showed that it is possible to perform mining on EHR data albeit with some challenges and limitations.

## Introduction

Rare genetic diseases affect 5–8% of the population and account for a disproportionately increased use of healthcare resources given the multisystemic manifestations^1,2^. However, these rare genetic diseases often go undiagnosed, with patients undergoing a prolonged diagnostic odyssey^3^. One such example is Fabry disease, which is a rare multisystemic genetic disease with a reported annual incidence of 1 in 100,000, although this is believed to be an underestimation as many cases remain undiagnosed^4^. There is a substantial time gap between the age of first symptoms and diagnosis: 9 and 23 years old for males and 13 and 32 years old for females, respectively^5^. Another example is familial hypercholesterolaemia (FH), which is a genetic disease resulting in greatly increased levels of low-density lipoprotein cholesterol (LDL-C). Such elevations in LDL-C lead to the premature development of numerous problems, such as angina and myocardial infarction^6^. Diagnosis is often delayed with patients only diagnosed after presenting with a catastrophic event^7^.

Electronic health record (EHR) describes how comprehensive patient data can be gathered between institutions and over a long period of time. Such a longitudinal and comprehensive amalgamation of data can go beyond the initial requirement of addressing medical treatment and provide invaluable information about a patient’s overall and general health^8^. EHRs contain rich volumes of patient data including demographics, medications, laboratory test results, diagnosis codes, and procedures, and while their primary use is to facilitate patient care, the existence of such data in a digitised format presents a unique opportunity for analysis and novel clinical insights, including identifying patients with rare genetic diseases. Indeed, Phenotype Risk Scores (PheRS) is a method to detect genetic disease patterns using phenotypes from EHR and has been shown to be a scalable method to find patients with undiagnosed genetic disease^9,10^.

While EHR data, in theory, can be considered to be standardised across different healthcare system, there is paucity of literature on such data mining approaches within the Asian healthcare system^11^, especially for rare genetic disorders. This could be partly be attributed to lack of EHR systems in many of the Asian healthcare systems, relying primarily on traditional paper based health records, but also due to challenges associated with infrastructural and skilled manpower constraints associated with implementation and data mining of EHR^12^. This study aims to be a preliminary step towards using EHR data to perform data mining to identify patients with an undiagnosed rare genetic disease, specifically Fabry disease and FH, as well as understand challenges in an Asian healthcare system context. This study is an early step towards increasing the quality of care for people living with rare disease by reducing their diagnostic odyssey so that more time and resources can be spent on managing the disease.

## Methods

### Data

This work was performed on a dataset consisting of deidentified structured medical records of approximately 1.28 million patients across three healthcare institutions under the Singapore Health Services (SingHealth) cluster in Singapore (National Heart Centre Singapore, KK Women’s and Children’s Hospital, and Singapore General Hospital) over a 3-year window (1 Jan 2018–1 Mar 2022). All methods and analysis were carried out in accordance with relevant guidelines and regulations and were approved by the SingHealth Data Governance committee. SingHealth Centralised Institutional Review Board has waived need for informed consent for this study.

### Data extraction, deidentification and preparation

The initial dataset was collected from various sources, including laboratory, radiology, pathology, diagnoses, and detailed patient information within the SingHealth Database, all of which contained identifiable patient information. Only structured data was extracted for this initial pilot (Supplementary Table 1), and free-text fields were excluded to minimise risk of exposing sensitive information. As a critical step towards ensuring privacy and compliance with security protocols, using a trusted third party, data fields deemed as sensitive were identified based on “SingHealth Policy for Data Anonymisation” and were pseudonymised. For example, the NRIC of patients was changed from “SxxxxxxxZ” format to “PIDxxxxxx” format. Such sensitive fields were initially randomly sorted and then PID numbers were assigned. After pseudonymisation, the data were then transferred to the Office of Insights and Analytics (OIA) High-Performance Computer Lab, which is an air-gapped environment. Strict security guidelines were in place to ensure that only users directly involved in the study were allowed access to the pseudonymised data. Such users were unable to transfer data onto or from the Lab devices themselves and required the permission and assistance of other Lab staff. The record linkage key was also not transferred into the lab and not exposed to the clinicians involved in this study by any means, hence, ensuring that the privacy of the individuals was not compromised.

Once deidentified, the structured data was then normalized and standardized using a third-party platform, Population Builder (Health Catalyst, USA). Population Builder tool allows the user to select multiple parameters and apply them as filters on the patient population (Supplementary Table 2). Problem list filtering was done with Systematized Nomenclature of Medicine Clinical Terms (SNOMED) codes. In many cases, multiple SNOMED codes refer to the same disease/phenotype in question (for example, familial hypercholesterolemia and familial hypercholesterolaemia). This poses a challenge when using such terms for searching through the data, in that every time one wants to apply a filter, all spelling and naming variations must be included. This problem was solved with the use of value sets. Value sets are a feature of the Population Builder tool, allowing for codes to be grouped into one unified set so that the user may simply add the set in the filtering process rather than manually searching for all the codes. Creating value sets requires the work to be done only once, then the set is readily available for the user to add to patient filtering whenever they please.

### Rare diseases

As a pilot, we selected two rare diseases for this project: Fabry Disease and Familial Hypercholesterolemia (FH). These diseases were selected as the diagnostic criteria for these two diseases are well defined and datapoints needed for the diagnostic criteria can be extracted or inferred from structured health records. The diagnostic criteria included:

1. Fabry disease

If patient is

- Less than 50 years old

AND has symptoms from AT LEAST two (2) of the following systems (broken down further into specific diseases).

- Kidney
  - Chronic kidney disease
  - Proteinuria
  - Microalbuminuria
- Cardiac
  - Cardiomyopathy
  - Valvular heart disease
  - Arrhythmia
- Neuro
  - Stroke ischemic
  - Transient ischemic attack
  - Acroparaesthesia
- Skin
  - Angiokeratomas
  - Impaired sweating/hypohidrosis
  - Heat and cold tolerance
- Eye
  - Corneal whirling
  - Cornea verticillate
  - Corneal and lenticular opacities
  - Vasculopathy (retina, conjunctiva)

(2) Familial hypercholesterolemia

If a patient has premature atherosclerotic cardiovascular disease (ASCVD) OR has severely elevated low-density lipoprotein calculated (LDL-C) laboratory results. More specifically if a patient satisfies any of the following.

- ASCVD, male, less than 55 years old
- ASCVD, female, less than 60 years old
- LDL-C greater than 2.6 mmol/L whilst adhering to high-intensity statins
- LDL-C greater than 3.9 mmol/L and 18 years old or younger
- LDL-C greater than 4.9 mmol/L and more than 18 years old

Patients who meet the criteria are flagged as a Fabry suspect or FH suspect, respectively^13,14^. We also created value sets for Fabry disease and FH (Supplementary Fig. 1) so as to identify patients with known diagnosis of these diseases within our database.

### Data wrangling

Specific metrics were examined based on each patient cohort. The data used for Fabry TPs and suspects included demographics (age, race, sex) and systems (which systems, from the diagnostic criteria, were affected for each patient) data. The data used for FH included demographics and LDL-C laboratory results. The data was retrieved from the database using Microsoft SQL Server Management Studio. Various SQL queries were written and executed, and the outputs were saved as CSV files to be loaded into RStudio. Once the CSV files were converted into R data frames, various manipulations were made in order to get the data into the correct shape and form for the various analyses that were performed (namely: visualisation, and statistical testing).

### Data analysis

As mentioned above, data analysis came in two forms for this project, and they are described below.

#### Visualisation

The tidyverse and lubridate R packages were used heavily in the visualisation portion of the project. Demographic data for both diseases was used to generate pie charts to visualise the race and sex breakdown of TPs and suspects for each respective disease. Age data for both diseases was used to generate scatterplots and boxplots (depending on the number of observations) showing the age distribution of male and female patients of each respective disease. Fabry disease systems data was used to create bar graphs for first, second, and third-order interactions and a five-way Venn diagram showing the interactions.

#### Statistical testing

A two-sample t-test for a difference in means was conducted on the LDL-C data for FH TPs and suspects using the following hypotheses:

##### H_0_:

 The mean LDL-C levels are equal between TP and suspect cohorts

##### H_A_:

 The mean LDL-C levels are different between TP and suspect cohorts

## Results

### Fabry disease

Out of the 1.28 million patients, only 4 patients (true positives, TPs) were identified to have a confirmed diagnosis of Fabry disease (Supplementary Fig. 2), giving a prevalence of 1 in 320,000. Assuming a prevalence of 1 in 100,000, this suggests there were potentially undiagnosed patients in our database. All 4 TPs were Chinese, consisting of 3 males and 1 female. The female TP was the youngest.

We then applied our criteria for Fabry disease and identified 2213 Fabry suspects. The suspects’ demographics are slightly more varied, due to the greater number of observations. Among the suspects, the male to female ratio is about 60:40 split; and, while the majority are Chinese (61%), there are small portions of other races present (15.5% Malay, 11.7% Indian, 11.8% others), which is consistent with the ethnic distribution in Singapore. The median age for both male and female suspects are both barely older than 40 years old, with the median male age slightly greater than that of females. Unfortunately, the small size of known cases of Fabry precluded any further comparisons with the suspect cases.

**Figure 1:**
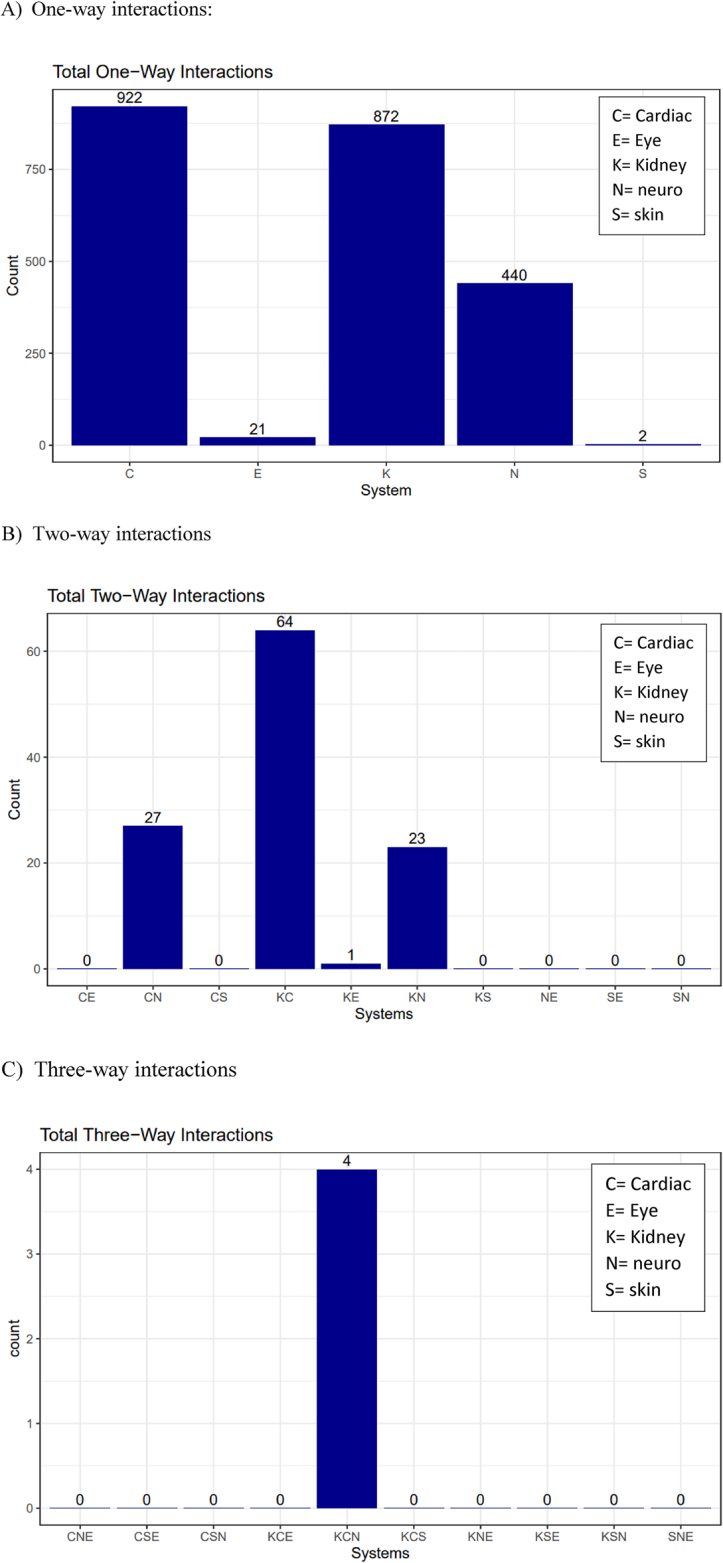
System interactions for suspect Fabry disease.

Given that patients with Fabry disease present with multisystemic involvement, we then reviewed the data pertaining to system interactions. Due to the multi-systemic nature of the disease, a patient may be in contact with multiple specialists at any given point in time, receiving care for the specific organ system. Examining affected systems data may provide insights into significant comorbidities for Fabry. The five systems involved in descending order of frequency are renal (922), cardiac (872), neurological (440), ophthalmologic (21), and cutaneous (2) (Fig. 1A). Exploring two-way interactions, we identified 64 cardiac-renal, 27 cardiac-neuro, 23 renal-neuro, and 1 renal-ophthalmologic interactions (Fig. 1B). Expanding to three-way interactions, we identified 4 patients with interactions across cardiac-renal-neuro system (Fig. 1C). Interrogating these 4 patients with 3 system interaction identified 2 potential cases who deserve further work up to exclude Fabry disease, while the other two had underlying comorbidity that could explain their 3-system interaction (Supplementary Text).

### Familial hypercholesterolemia

The TP cohort for FH is substantially larger than that of Fabry, with 161 confirmed diagnoses, giving us a prevalence of 1 in 8000. In contrast, based on a recent genomic epidemiology study of Singaporeans^15^, the population prevalence of FH in our local population is estimated to be 1 in 250, suggesting that we were severely underdiagnosing FH in our system. Using our screening criteria, we identified 12,328 FH suspects. There was a slight difference in the gender breakdown between TPs and suspects. TPs have about a 53:47 male to female ratio whereas suspects have a 42:58 ratio. On average, female TPs were older than male TPs, and the same is true for female and male suspects, with more variability in the TPs (greater interquartile range (IQR)).

**Figure 2:**
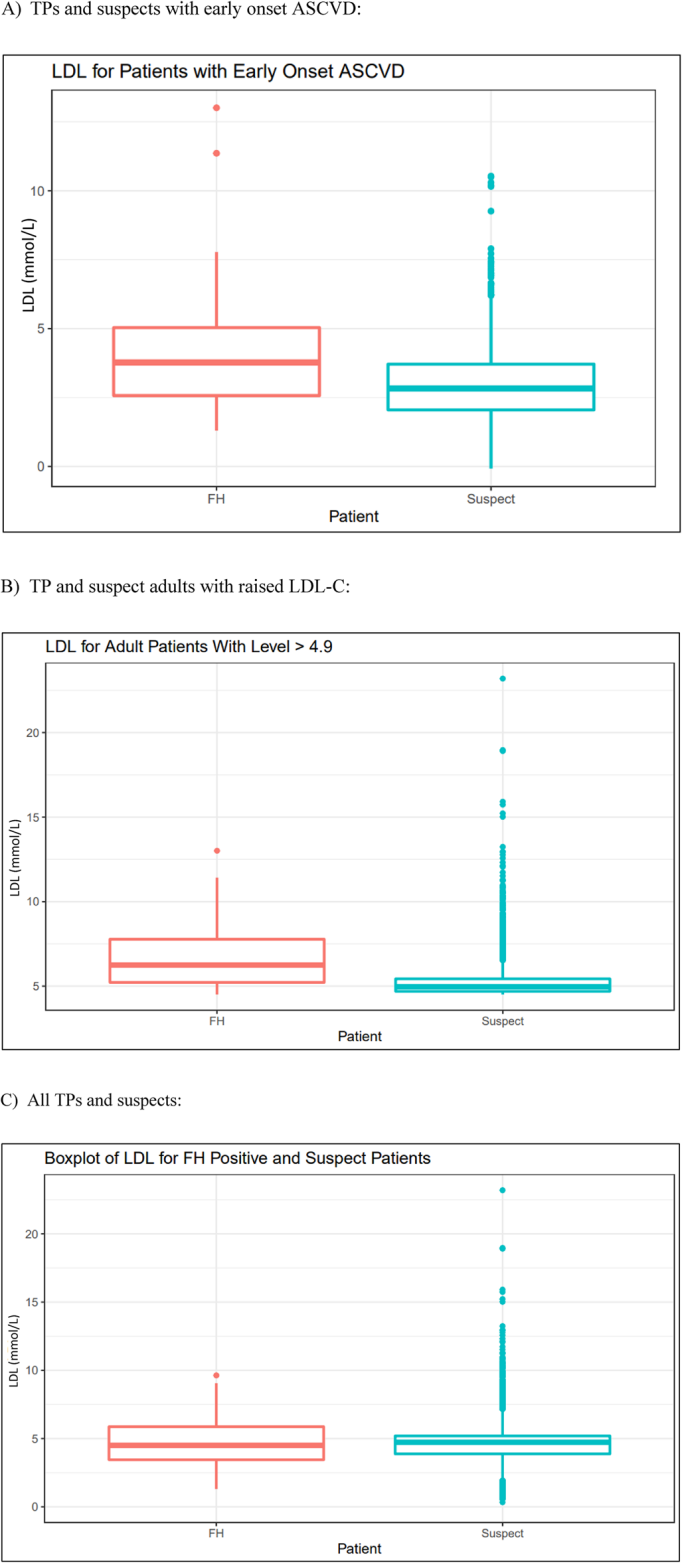
Boxplots for patients with known FH and suspect FH.

Laboratory results provide good numeric data about patients. LDL-C is used in the diagnostic criteria; thus, these lab results were extracted for analysis. The TP and suspect cohorts were split into the main diagnostic criteria for graphing. The median LDL-C level for TPs satisfying the ‘early onset ASCVD’ red flag is slightly greater than that of the suspects (Fig. 2A). The same is true for patients satisfying the ‘adult with raised LDL-C’ red flag; the data for suspects in this subset is right-skewed, with most of the incidences being very close to the red flag level of 4.9 (Fig. 2B). We also observed some extreme values, with one patient’s LDL-C well over 20 mmol/L. Grouping all the data together into TP and suspect sets, it can be seen that the median LDL-C value for TPs is very close to that of the suspects (p-0.1022) (Fig. 2C).

## Discussion

Our proof-of-concept study, spanning 1.28 million records, identified 2 potential patients with Fabry disease, leading to a potential 50% increase in the number of patients with Fabry disease within our healthcare system. In addition, we identified over 12,000 participants with suspected FH, which is closer to expected prevalence of FH in our population, suggesting that patients with rare genetic diseases such as Fabry and FH are often underdiagnosed. This presents as a missed opportunity as early identification and treatment is associated with better healthcare outcomes^16,17^.

Within the current literature, there are multiple examples of data mining successfully applied to EHR data, in fields such as: “Understanding the Natural History of Disease”, “Cohort Identification”, “Risk Prediction/Biomarker Discovery”, “Quantifying the Effect of Intervention”, “Constructing Evidence-Based Guidelines”, and “Adverse Event Detection” ^18^. Denny described how EHR data can be used for genomic analysis and discussed various challenges accompanying this task, including missing data, incorrect data, and unstructured data^19^. Jensen et al. also described the issue of unstructured text data in EHRs, pointing out that improvements in text mining techniques were making these parts of the EHR more accessible for data mining^20^. Kirk et al. implemented a text-mining approach to match patient records with two clinical vocabularies to perform data clustering on 14,017 patients^21^. In addition to clinical features, administrative factors such as length of stay in the hospital have been examined with the aid of EHR data as well^22^. More recently, Landi et al. demonstrated that the vast quantities of data within EHRs presented the opportunity for deep learning to be applied. Deep-learning is a method of machine learning, which uses neural networks to tease out information from data^23^. Liang et al. identified the promise, albeit underdeveloped, of EHRs to assist with the identification of interactions between co-existing morbidities, such as severity of COVID-19 in patients with immunodeficiency^24^. There are many steps involved in the process of rendering EHR data usable for mining. As such, there are many stages of the process which can be improved using technology. Both structured and unstructured (data in the form of free text) EHRs can be improved by a search engine to assist in finding patients; natural language processing can be applied to identify phenotypes; machine learning can be applied to classify patients^25^.

One of the biggest issues with such approaches is the underlying dataset, which can vary across healthcare systems and present with their own unique set of challenges, and the algorithm needs to be modified accordingly to address those challenges. One of the purposes of this proof-of-concept study was to show what can be done on such a dataset from an Asian healthcare setting.

While the study shows the potential utility of flagging potential cases, it is not possible to confirm the diagnosis in those specific patients, because the data has been deidentified irreversibly. Indeed, this is a limitation to our study as we are unable to confirm or refute the diagnosis in patients that were flagged for further review. However, to add a layer of additional validation, we had manually reviewed the deidentified medical records of all the flagged patients and considered them as potential cases only if they fulfilled the clinical criteria and did not have a more plausible secondary cause for their multi-organ manifestation (supplementary text). For example, individual PIDxxx4 with chronic kidney disease, transient ischemic attacks and cardiomyopathy may have hypertensive nephropathy rather than Fabry disease, but this is outside the scope of this current study. However, our data suggests that such results can be used to guide decision-making and provide evidence for or against diagnostic criteria. It is also important to note that these results cannot be used in isolation to deliver definitive diagnosis and must be interpreted within clinical context and in partnership with domain-specific medical expertise.

Although substantial and useful in supporting proof-of-concept, another limitation of our study is that the data used for this project was limited to a 3-year window. This resulted in some patients presenting as TPs for a disease, but no other information about their state prior to/following their diagnosis was available for further context exploration. This is a problem because multiple data fields are required to implement any sort of statistical testing or machine learning. Such data censoring can be overcome by lengthening the window during which data is collected, but that is outside of the parameters of our current accessible data and can be assessed prospectively with this data set as it accumulates with time. In addition, the testing and fine-tuning of diagnostic criteria are critical. The data mining performed in this project was based on algorithms and diagnostic criteria supplied by subject matter experts. While such criteria are derived from relevant literature and have proven validity, care should be taken to ensure their statistical validity.

Phenotype risk scores (PheRS) is the sum of clinical features observed in an individual based on phecodes, a map of human phenotype ontology (HPO) terms mapped to billing codes within the EHR, weighted by the log inverse prevalence of the feature^10^. This approach has been shown to be a strong predictor of case status in cohorts of patients with rare diseases and assists with augmentation of rare variant interpretation and identification of rare disease patients with symptoms overlapping with common diseases^10^. However, our dataset was limited to structured data, which did not allow us to generate phecodes, and hence, we were unable to compare our strategy against PheRS in flagging potential cases within our dataset.

Lastly, while we did identify potential undiagnosed patients, this approach does not identify all such patients. Indeed, while we identified 2 potential patients with Fabry disease, bringing our total to 6, we would expect between 13 and 30 patients with Fabry disease in our dataset, suggesting that alternative complementary approaches will be required to comprehensively identify all the unidentified patients. However, while our single solution does not solve this complex problem, it offers a unique solution in addressing one element of this healthcare challenge.

This study was a pilot study, a proof of concept, with the goal to test whether EHR data contained enough/suitable data for data mining techniques to be applied. In the specific use case of rare diseases, not as many techniques as initially thought could be carried out. By nature, rare diseases will be very sparse in large patient datasets, especially when drilling down to one very specific disease, such as Fabry Disease. Given enough time or with a broader dataset, as more patients are diagnosed with rare diseases, the data could be built up and more algorithms could be applied. In addition, extracting unstructured data and extracting relevant HPO terms or phecodes could allow us to test the utility of other data mining approaches.

Potential expansion from such studies could include providing clinicians with pre-trained algorithms to automatically flag patients in their system that they may be at-risk of having a rare disease. Such patients can then be referred to genetic testing. If such a tool was effective at identifying potential rare disease cases, then the algorithm would be able to be trained on more and more data, both structured and unstructured, as time goes on, increasing its utility. An ideal end-product would be a computer application available to primary care practitioners during patient consultations, which have access to EHR data and can raise a warning when the application detects a rare disease ‘red-flag’ from either a pre-determined or dynamic data mining process.

## Conclusion

This project has shown that conducting mining on EHR data is possible. Even in de-identified, sparse, censored data, many useful insights were found. Directions and guidance on what to look for are crucial in tasks like these; as such, continuous and clear communication with subject matter experts is paramount. A wider variety of data fields could be included in analyses to improve on the work outlined in this project. The attributes used in our project were selected based on time constraints and data availability. By showcasing the opportunities and limitations of such a dataset, this project helps to strategize future work in the area.

### Supplementary Information

Supplementary Figures.

Supplementary Table S1.

Supplementary Table S2.

Supplementary Information 4.

## Data Availability

The data used in this study are not publicly available due to privacy and legal restrictions but are available from the corresponding author on reasonable request.
