# Author Correction: Analysis and visualisation of electronic health records data to identify undiagnosed patients with rare genetic diseases

**DOI:** 10.1038/s41598-024-60776-2

**Published:** 2024-05-02

**Authors:** Daniel Moynihan, Sean Monaco, Teck Wah Ting, Kaavya Narasimhalu, Jenny Hsieh, Sylvia Kam, Jiin Ying Lim, Weng Khong Lim, Sonia Davila, Yasmin Bylstra, Iswaree Devi Balakrishnan, Mark Heng, Elian Chia, Khung Keong Yeo, Bee Keow Goh, Ritu Gupta, Tele Tan, Gareth Baynam, Saumya Shekhar Jamuar

**Affiliations:** 1 Curtin University, Perth, Australia; 2 Health Catalyst, Utah, USA; 3 Genetics Service, Department of Paediatrics, KK Women’s and Children’s Hospital, 100 Bukit Timah Road, Singapore, 229899 Singapore; 4 SingHealth Duke-NUS Genomic Medicine Centre, Singapore, Singapore; 5 Department of Neurology, National Neuroscience Institute (Singapore General Hospital), Singapore, Singapore; 6 Department of Internal Medicine, Singapore General Hospital, Singapore, Singapore; 7 SingHealth Duke-NUS Institute of Precision Medicine, Singapore, Singapore; 8 Cancer & Stem Cell Biology Program, Duke-NUS Medical School, Singapore, Singapore; 9 Laboratory of Genome Variation Analytics, Genome Institute of Singapore, Singapore, Singapore; 10 National Heart Centre Singapore, Singapore, Singapore; 11 SingHealth Office of Insights and Analytics, Singapore, Singapore; 12 Data Analytics Office, KK Women’s and Children’s Hospital, Singapore, Singapore; 13 Rare Care Centre, Perth Children’s Hospital, Perth, WA Australia; 14 Western Australian Register of Developmental Anomalies, Perth, WA Australia

Correction to: *Scientific Reports* 10.1038/s41598-024-55424-8, published online 01 March 2024

The original version of this article contained an error in the title of the paper, where the term “Cluster” was erroneously added.

In addition, in the abstract,

“Data mining in the form of cluster analysis and visualisation, was performed on a database containing deidentified health records of 1.28 million patients across 3 major hospitals in Singapore, in a bid to improve the diagnostic process for patients who are living with an undiagnosed rare disease, specifically focusing on Fabry Disease and Familial Hypercholesterolaemia (FH).”

now reads:

“Data analysis in the form of visualisation and statistical testing, was performed on a database containing deidentified health records of 1.28 million patients across 3 major hospitals in Singapore, in a bid to improve the diagnostic process for patients who are living with an undiagnosed rare disease, specifically focusing on Fabry Disease and Familial Hypercholesterolaemia (FH).”

The original article has been corrected.

