# Supplementary material for: Analysis and visualisation of electronic health records data to identify undiagnosed patients with rare genetic diseases
Source: Sci Rep. 2024 Mar 1;14:5056. doi: 10.1038/s41598-024-55424-8 (PMC10904843; doi:10.1038/s41598-024-55424-8)

**SUPPLEMENTARY FIGURES**

**Supplementary Figure 1:** Value set for Fabry Disease

**
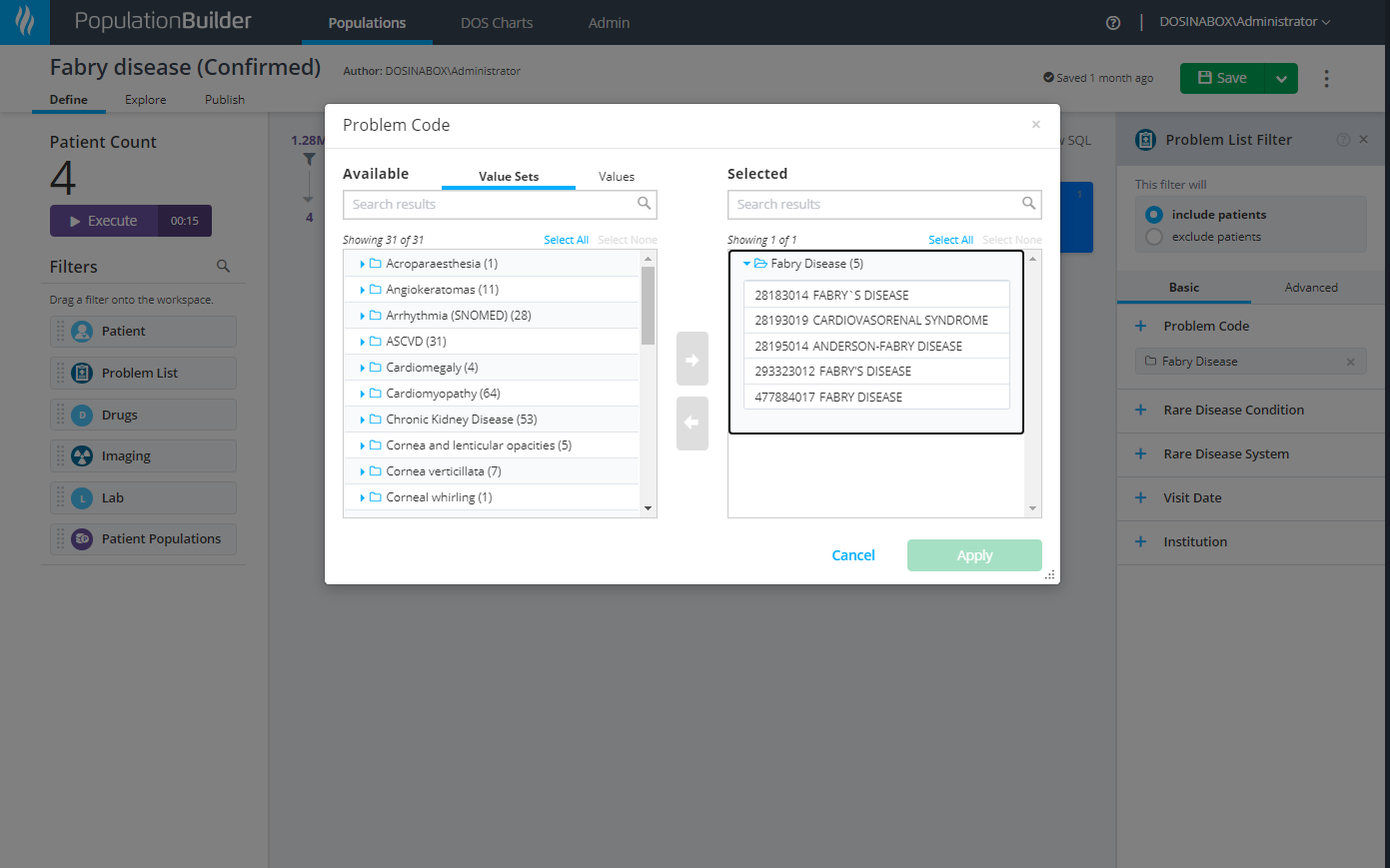
**

**Supplementary Figure 2:** Filtering using Health Catalyst’s Population Builder tool allowed filtering of 1.28 million patients using the Fabry disease screening criteria


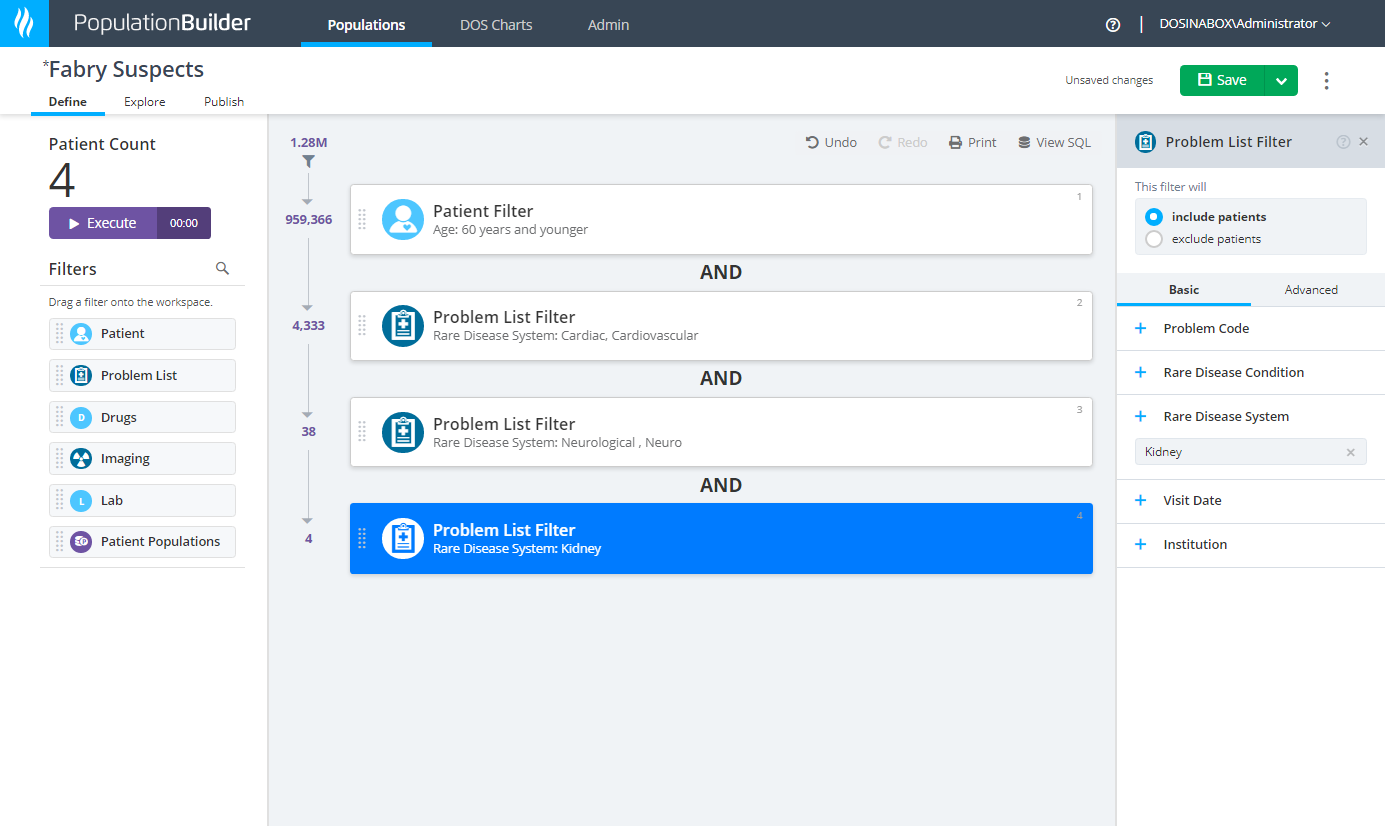

Supplement: Supplementary file 1 — Supplementary Figures. [file 41598_2024_55424_MOESM1_ESM.docx]
