# Supplementary material for: Analysis and visualisation of electronic health records data to identify undiagnosed patients with rare genetic diseases
Source: Sci Rep. 2024 Mar 1;14:5056. doi: 10.1038/s41598-024-55424-8 (PMC10904843; doi:10.1038/s41598-024-55424-8)
Supplement: Supplementary file 2 — Supplementary Table S1. [file 41598_2024_55424_MOESM2_ESM.docx]

**Supplementary Table 1: List of data fields extracted from EHR**

| **Dataset** | **Fields** |
| --- | --- |
| **Medication (Drug Dispensed And Ordered Subject)** | Patient ID |
|  | Race |
|  | Gender |
|  | Date of Birth |
|  | Visit No |
|  | Case No |
|  | Age |
|  | Admit / Visit Date |
|  | Item Code (Dispensed) |
|  | Item Description (Dispensed) |
|  | Dispensed Count (Dispensed) |
|  | Item Code (Ordered) |
|  | Item Description (Ordered) |
|  | Order Count (Ordered) |
|  | Institution Code |
| **Problem / SNOMED Code (Problem List Subject)** | Patient ID |
|  | Race |
|  | Gender |
|  | Date of Birth |
|  | Case No |
|  | Visit No |
|  | Institution |
|  | Visit Date |
|  | Case Type Desc |
|  | Age |
|  | Created Date |
|  | Problem Code (Coded) |
|  | Problem Desc (Coded) |
|  | Coding Scheme (Coded) |
|  | Problem Status |
| **Lab Test (Lab General Subject)** | Patient ID |
|  | Race |
|  | Gender |
|  | Date of Birth |
|  | Age |
|  | eHIntS Case Id |
|  | Case No |
|  | Specimen Collection Date |
|  | Service Description (Free Text) |
|  | Service Code (Free Text) |
|  | Service Description |
|  | Service Code |
|  | Verify Date |
|  | Lab Resulted Order Test Type (KKH Only) |
|  | Lab Resulted Order Test Code |
|  | Lab Resulted Order Test Description |
|  | Result Value |
|  | Reference Ranges |
|  | Reference Ranges Normal High |
|  | Reference Ranges Normal Low |
|  | Reference Ranges Critical High |
|  | Reference Ranges Critical Low |
|  | Abnormal Flags |
|  | Result Comments |
|  | Units of Measurements |
|  | Institution Code |
| **X-ray (Radiology Subject Area)** | Patient ID |
|  | Race |
|  | Gender |
|  | Date of Birth |
|  | Patient Age |
|  | eHIntS Visit Id |
|  | Updated Date Time |
|  | Procedure Name |
|  | Result Item Code |
|  | Obs Item Name |
|  | Line Num |
|  | Text |
|  | Institution Code |
| **Histology (Lab Patho Subject)** | Patient ID |
|  | Race |
|  | Gender |
|  | Date of Birth |
|  | Entry Date |
|  | eHIntS Case Id |
|  | Case No |
|  | Service Code (Free Text) |
|  | Service Description (Free Text) |
|  | Service Code |
|  | Service Description |
|  | Tissue Description |
|  | Tissue Mnemonic |
|  | T Code |
|  | Final |
|  | Institution Code |
