# Supplementary material for: Analysis and visualisation of electronic health records data to identify undiagnosed patients with rare genetic diseases
Source: Sci Rep. 2024 Mar 1;14:5056. doi: 10.1038/s41598-024-55424-8 (PMC10904843; doi:10.1038/s41598-024-55424-8)
Supplement: Supplementary file 3 — Supplementary Table S2. [file 41598_2024_55424_MOESM3_ESM.docx]

**SUPPLEMENTARY TABLE 2: Filters on Population Builder**

- Patient
  - Age
  - Gender
  - Race
- Problem List
  - Problem code
  - Problem description
- Labs
  - Lab description
  - Value (greater than/less than)
  - Date
- Drugs
  - Drug description
  - Dispensed/ordered
- Imaging
  - Presence of phenotypes (Y/N)
