# Supplementary material for: Analysis and visualisation of electronic health records data to identify undiagnosed patients with rare genetic diseases
Source: Sci Rep. 2024 Mar 1;14:5056. doi: 10.1038/s41598-024-55424-8 (PMC10904843; doi:10.1038/s41598-024-55424-8)
Supplement: Supplementary file 4 — Supplementary Information 4. [file 41598_2024_55424_MOESM4_ESM.docx]

| **Patient ID** | **Race** | **Age (in years)** | **Gender** |
| --- | --- | --- | --- |
| PIDxxx1 | Chinese | 49 | Female |
| PIDxxx2 | Chinese | 47 | Male |
| PIDxxx3 | Chinese | 54 | Male |
| PIDxxx4 | Indian | 56 | Female |

Patient PIDxxx2 is a 47 year old Chinese male. Review of his medical data revealed that the individual has chronic kidney disease, arrhythmogenic right ventricle cardiomyopathy and a history of ischemic stroke. He did not have any other secondary diagnoses such as Diabetes Mellitus, familial hypercholesterolemia, etc. that could explain the presence of these three diagnoses, and is an individual who would benefit from further screening for Fabry disease.

Patient PIDxxx4 is a 57 year old Indian female with chronic kidney disease, transient ischemic attack and cardiomyopathy. This individual, like patient PIDxxx2, would benefit from further investigations for Fabry disease.

In contrast, patient PIDxxx1 is a 49 year old Chinese female, who has diagnoses of end stage renal failure, history of ischemic stroke and cardiomyopathy. However, she also has a diagnosis of adult onset polycystic kidney disease, which is the likely cause of her other medical diagnoses, and hence, this individual would not benefit from further investigations for Fabry disease.

Patient PIDxxx3 is a 54 year old Chinese male with dilated cardiomyopathy, end stage renal failure and neurological symptoms of altered mental status and giddiness. However, the individual also had diabetes mellitus and hypertension, and his neurological phenotype was not consistent with Fabry disease. Hence, this individual would not benefit from further investigations for Fabry disease.
